# Prodigiosin inhibits motility and activates bacterial cell death revealing molecular biomarkers of programmed cell death

**DOI:** 10.1186/s13568-016-0222-z

**Published:** 2016-07-26

**Authors:** N. Darshan, H. K. Manonmani

**Affiliations:** 1 Academy of Scientific and Innovative Research (AcSIR), New Delhi, 110 025 India; 2 Department of Food Protectants & Infestation Control, Central Food Technological Research Institute (CSIR–CFTRI), Mysore, 570 020 India

**Keywords:** Prodigiosin, *Serratia nematodiphila*, Bactericidal activity, Programmed cell death, Motility

## Abstract

The antimicrobial activity of prodigiosin from *Serratia nematodiphila darsh1*, a bacterial pigment was tested against few food borne bacterial pathogens *Bacillus cereus*, *Staphylococcus aureus*, *Pseudomonas aeruginosa* and *Escherichia coli*. The mode of action of prodigiosin was studied. Prodigiosin induced bactericidal activity indicating a stereotypical set of biochemical and morphological feature of Programmed cell death (PCD). PCD involves DNA fragmentation, generation of ROS, and expression of a protein with caspase-like substrate specificity in bacterial cells. Prodigiosin was observed to be internalized into bacterial cells and was localized predominantly in the membrane and the nuclear fraction, thus, facilitating intracellular trafficking and then binding of prodigiosin to the bacterial DNA. Corresponding to an increasing concentration of prodigiosin, the level of certain proteases were observed to increase in bacteria studied, thus initiating the onset of PCD. Prodigiosin at a sub-inhibitory concentration inhibits motility of pathogens. Our observations indicated that prodigiosin could be a promising antibacterial agent and could be used in the prevention of bacterial infections.

## Introduction

Programmed cell death (PCD) is a genetically encoded mechanism that leads to cell death, PCD in bacteria is always a debatable topic. The existence of PCD like bacterial death suggests a significant conceptual change in our understanding of bacteria. PCD as a target for antibacterial therapy will be important for revealing the death pathways involved, we were able to unravel the mechanism underlying this bacterial programmed cell death. Historically, bacteria have been the responsible for some of the most deadly diseases and widespread epidemics of human civilization. Bacterial infectivity results from disarray in the balance between bacterial virulence and host resistance. The number of qualities has been determined that may contribute to pathogenicity including swarming motility, swimming and extracellular enzyme activities, i.e. protease, nuclease, and hemolysin (Hejazi and Falkiner 1997). The fight against bacterial infection is one of the great success stories of medicinal chemistry and pharmacology. Ever since the discovery of antibiotics, plenty of bactericidal and bacteriostatic chemicals have been available. In the past 60 years, antibiotics have been vital in the fight against infectious diseases caused by bacteria and other microbes. However, antibiotic resistance has become a serious and growing concern in modern medicine and has emerged as one of the pre-eminent public health problems of the 21st century. Hence, the demand for new antibiotics with specific molecular targets continues to grow due to the antibiotic-resistant pathogens causing life-threatening infections in spite of considerable advancement in the fields of chemical synthesis and engineered biosynthesis of antimicrobial compounds. This changing scenario of bacterial diseases continuously demands the discovery of novel antibiotics/antimicrobial compounds while addressing drug resistance. Prodigiosin is reported to be a very interesting alternative with antibacterial activity. Although prodigiosin has no known distinct and defined role in the physiology of the strains in which it is produced, it has antifungal, antiproliferative and antiprotozoal activities, and thus may have potential clinical utility (Moraes et al. 2009). This paper addresses the bacteriostatic/bactericidal activity of prodigiosin from *Serratia nematodiphila* emphasizing on the programmed cell death like activity against some selected foodborne bacterial pathogens.

## Materials and methods

### Isolation and identification of *S. nematodiphila*

For isolation of *S. nematodiphila*, soil sample were collected from the agricultural farm in Mandya district, Karnataka, India, the red pigment producing bacteria were isolated using pour plate method on tryptic soy agar (tryptone15 g/L; soytone—enzymatic digest of soybean meal 5 g/L; sodium chloride 5 g/L; agar 15 g/L; pH7.0).Red coloured colonies were selected and subcultured on the same medium until pure colonies were obtained. Purified cultures were preserved at −20 °C in 50 % (v/v) glycerol. Physiological and biochemical characters of the bacterial isolate were examined according to methods described in Bergey’s manual of systematic bacteriology (John 1994). The strain was characterized by using 16S ribosomal RNA gene and has been deposited in the GenBank with an accession number KU886227, The culture was deposited as *S. nematodiphila darsh1* with deposition number NCIM 5606.

### Screening for prodigiosin production

The bacteria were streaked onto glycerol-Peptone Agar (agar 15 g/L; glycerol 10 g/L; peptone 10 g/L; pH7.0) plates and incubated at 30 °C for 24 h. The cells were washed in the 1X phosphate buffer saline (137 mM NaCl; 2.7 mM KCl; 8 mM Na_2_HPO_4_; 2 mM KH_2_PO_4_; PBS 7.2) and collected by centrifugation at 10,000 rpm for 10 min. The washed cell pellet was resuspended in acidified methanol, vortexed for 15 min and subjected to centrifugation at 10,000 rpm for 10 min thrice at 28 °C. The cell-free supernatant was taken in two test tubes. The first tube content was acidified with concentrated HCl, whereas, the other was alkalinized with concentrated ammonia solution. A red or pink colour obtained in acidified solution and a yellow or tan colour in the alkaline solution indicated a positive, presumptive test for prodigiosin (Gerber and Lechevalier 1976).

### Extraction and purification of prodigiosin

The crude prodigiosin was extracted from cell-free supernatant using acidified methanol. Prodigiosin was concentrated using a rotary evaporator (Buchi, Flawil, Switzerland) and the concentrate obtained was dissolved in acidified methanol (96 ml ethanol and 4 ml HCl). The resulting solution was passed through a hexane-balanced silica gel column (mesh size 80–100), and the reddish orange fraction was eluted out. This fraction was dried in a vacuum oven at 45 °C to obtain the purified prodigiosin.

### Quantification of prodigiosin

The bacterial cells suspended in Phosphate buffered saline (PBS pH 7.2) and cell-free supernatant were subjected to spectrum scanning in the range of 300–700 nm using a UV–VIS spectrophotometer (Shimadzu UV 1800) and acidified methanol was used as a blank. Extracted prodigiosin was estimated as per the following equation (Slater et al. 2003).

$${\text{Prodigiosin}}\;{\text{unit/cell}} = \left[ {{\text{OD}}_{499} {-} \left( {1.381} \times {\text{OD}_{620} } \right)} \right] \times {1000/{\text{OD}}_{620}}$$

where, OD_499_—pigment absorbance, OD_620_—bacterial cell absorbance, 1.381—constant

Prodigiosin auto fluorescence was measured at an excitation of 543 nm and an emission of 570 nm and quantified in comparison to the standard commercial prodigiosin purchased from adipogen (USA) used as the standard.

### Identification of prodigiosin

The purified prodigiosin was dissolved in methanol and syringe filtered (0.2 µm) immediately before HPLC analysis. Chromatographic separation was carried out using an RP-18 column for isocratic chromatography (Shimadzu LC-8A, 5 µm, 18 × 100 mm), with a flow rate of 1 ml min^-1^ and an injection volume of 10 µL. The solvents used were methanol/10 mM triethylamine (19/1, v/v). The wavelength for detection was 533 nm. The concentration of prodigiosin was identified by measuring the absorbance, and then calculated using a standard correlation curve between absorbance and the dry weight of prodigiosin.

### Characterisation by LC–MS and ^1^H-NMR analysis

The characterization of the purified prodigiosin was done by LC–MS (Waters Quattro Premier Micromass) and ^1^H NMR (Bruker NMR 400 MHz). Prodigiosin dissolved in methanol was injected into the LC–MS and MS was performed using positive ion electrospray ionization with the following settings: capillary voltage 3.4 V, cone voltage 30 V at a source temperature 100 °C.

Prodigiosin dissolved in d-chloroform was analysed by ^1^H NMR to identify and confirm the structure of the purified product.

### Antibacterial activity

The antibacterial activity of prodigiosin was tested against *Bacillus cereus* (MTCC 1272), *Staphylococcus aureus* (MTCC 96), *Pseudomonas aeruginosa* (Lab isolate, DT CT1) and *Escherichia coli* (MTCC 729) by the Kirby–Bauer disc diffusion method. Sterile discs (Whatmann filter paper No. 1) were soaked with 50 µg/ml of prodigiosin dissolved in methanol, air dried under sterile conditions. These discs were mounted on nutrient agar (agar 15 g/L; peptone 5 g/L; beef extract 3 g/L; NaCl 5 g/L; pH7.0) plates, previously spread plated with the target bacterial isolate at a concentration of 10^6^ CFU/ml. Disc soaked with methanol and standard antibiotic discs were used as a control and for comparison respectively. The plates were incubated at 30 °C for 24 h. Antibacterial activity was expressed in terms of the diameter (mm) of the zone of inhibition formed around the discs.

### Determination of minimum inhibitory concentration (MIC) and minimum bactericidal concentration (MBC)

The MIC and MBC of purified prodigiosin against these pathogenic strains were tested. The pathogens were grown in nutrient broth (peptone 5 g/L; beef extract 3 g/L; NaCl 5 g/L; pH7.0) supplemented with increasing concentrations of prodigiosin (1–50 µg/mL) in 96 well micro titre plates and incubated at 30 °C for 24 h. A positive (cell treated with ciprofloxacin; 1 mg/mL) and negative control (cells without any drug) well was included for every test bacteria to validate adequate microbial growth over the course of the incubation period. The plate was analysed for turbidity using Varioskan Flash Multimode Plate Reader (Thermo Scientific) after 24 h of a growth period. The MIC was defined as the lowest concentration of prodigiosin that resulted in the complete inhibition of bacterial growth under study. The MBC was determined by plating samples from the treated wells (as given above) with concentrations above the MIC on to fresh plates of nutrient agar and counting viable CFU/ml. The MBC is the lowest concentration that demonstrated a preset reduction (such as 99.9 %) in CFU/ml when compared to the MIC dilution.

### Mode of bacteriostatic/bactericidal action of prodigiosin on foodborne pathogens

The following representative potent foodborne pathogen *E. coli* and *B. cereus* were chosen randomly out of the tested four pathogens to deduce the mode of action of prodigiosin by the following methods.

### Measurement of reactive oxygen species (ROS) in prodigiosin treated bacterial cells

The pathogens were grown in nutrient broth supplemented with 50 µg/ml prodigiosin in 96 well microtiter black plates and incubated at 30 °C for 24 h. The cells were harvested by centrifugation at 4000 rpm for 10 min, at 4 °C. Reactive oxygen species (ROS) generation was measured using 2, 7-dichlorofluorescin diacetate (DCFH-DA) dye. The cell pellet was incubated with 100 μM DCFH-DA at 37 °C for 45 min under dark. Measurement of fluorescence was done using a Varioskan Flash Multimode Plate Reader (Thermo Scientific) with excitation and emission wavelengths of 485 and 520 nm, respectively.

### Scanning electron microscopy (SEM) of prodigiosin treated pathogens

SEM was used to evaluate the morphological changes in the pathogens prior to and after treatment with prodigiosin, Prodigiosin (50 µg/ml) was added to the test organism and were then incubated for 2, 6, 18 and 24 h at 37 °C. The treated pathogen cultures collected at each time interval were centrifuged at 6000 rpm for 15 min to collect the cell pellet. The cell pellet was washed twice with potassium phosphate buffer (0.01 M; pH 7.0) followed by overnight fixing of the pellet with 2 % glutaraldehyde. Then, the pellet was dehydrated in ethanol on a gradient mode (10–100 %). Upon completion of the wash with 100 % ethanol, the pellet was spread on to the previously sterilized slide as a thin uniform layer and the slides were kept in a desiccator for drying. The slides were then subjected to SEM studies using an SEM (LEO 435VP, UK) attached to a video copy processor (Mitsubishi, Japan). The amplified image was photographed by a 35 mm camera (Ricoh, Japan).

### Analysis of phosphatidylserine exposure

Annexin V labelling of prodigiosin treated pathogens was performed using Annexin V-FITC Apoptosis Detection Kit (Sigma) (Dwyer et al. 2012). Propidium iodide was used as a counterstain for identification of dead cells. The protocol is followed according to the kit manufacturer. The fluorescence of the incubated cells was checked to detect phosphatidylserine exposure, with a flow cytometer (Beckmancoulter cell lab quanta SC).

### Analysis of depletion of the lipopolysaccharide layer

Prodigiosin treated pathogens were suspended in staining buffer (phosphate-buffered saline, 1 mM EDTA, tween-20, 0.1 % sodium azide, pH 7.4). This suspension (0.2 ml) was vortexed, incubated with 17 μM thiazole orange solution (5 µl) and 1.9 mM of ethidium bromide solution (5 µl) for 5 min. The flow cytometer analysis was carried out to determine the depletion of the lipopolysaccharide layer (Nebe-von-Caron et al. 2000). The cells after prodigiosin treatment were resuspended in binding buffer (100 mM NaOH; 1.4 M NaCl; 25 mM CaCl_2_; pH 7.5) and TO and PI were added at a final staining concentrations of 420 nM for TO and 48 µM for PI, and cells were analysed using flow cytometer (Beckmancoulter cell lab quanta SC).

### Motility test for prodigiosin treated pathogens

To assess bacterial motility in the presence or absence of sub inhibitory concentrations of prodigiosin the well-established TTC based soft agar (beef extract 3 g/L; pancreatic digest of casein 10 g/L; sodium chloride 5 g/L; agar 4 g/L; pH 7.0) method was used. Equal numbers of pathogenic bacteria grown in nutrient broth were inoculated into semi-solid medium containing up to 1 % TTC by stabbing the centre of the column of medium to greater than half the depth into tubes. Bacterial migration through the semi-solid agar was assessed after incubation (24 h). Motility is observed as diffused growth away from the stab inoculation line while non-motile organisms grow along the stab line.

### Analysis of expression of a protein with caspase-like substrate specificity

The expression of specific protease with caspase-like substrate specificity that could bind peptides encoding caspase substrate sequences Asp-Glu-Val-Asp (DEVD) and cleaves a number of different bacterial proteins was detected using EnzChek^®^ Caspase-3 Assay Kit from molecular probes by measuring the fluorescence (excitation/emission ~496/520 nm) using (Varioskan Flash Multimode Plate Reader, Thermo Scientific). We attempted to determine experimentally if functional orthologs exist in these pathogens by searching for bacterial proteins those with the ability to bind synthetic caspase substrate peptides (Garcia-Calvo et al. 1998) following prodigiosin treatments that might induce bacterial cell death.

### DNA fragmentation assay

The pathogens were treated with Prodigiosin (50 µg/ml) and incubated in lysis buffer (20 mM Tris pH 8; 20 mM EDTA; 1 % Triton X-100 and 20 mg/ml of lysozyme at pH 8) for 2 h at 37 °C. The cell lysate was centrifuged at 10,000 rpm for 15 min. The supernatant was treated with proteinase K (0.2 mg/ml) in a buffer (150 mM NaCl; 10 mM Tris–HCl pH 8.0; 40 mM1 % SDS and EDTA) for 4 h at 37 °C. DNA was isolated with phenol/chloroform and precipitated using ice cold alcohol. DNA pellets were resuspended in TE buffer (10 mM Tris–HCl pH 8.0; 1 mM EDTA) and treated with DNase-free RNase for 1 h at 37 °C. Finally, samples were electrophoresed in a horizontal 1 % agarose gel and stained with ethidium bromide to visualize under UV illumination.

### Mechanism of DNA binding

#### Absorbance and fluorescence spectra measurements

Absorbance spectra were measured using a UV/Vis spectrophotometer (Shimadzu UV1800). Fluorescence measurements were performed with a using a multi-well reader (Varioskan Flash Multimode Plate Reader, Thermo Scientific). Corrected excitation and emission spectra of prodigiosin were obtained both in buffer alone and in the presence of excess lambda DNA, in order to obtain spectra for the free and bound forms. For prodigiosin, excitation spectra were measured with λ_ex_ = 543 nm, while emission spectra were measured with λ_em_ = 570 nm. λDNA (Merck Genei) sufficiently free of protein was used. A260/A280 of 1.8 was considered for the assay. DNA concentrations were determined spectrophotometrically (Eppendorf bio spectrophotometer Basic). All the experiments were carried out in 5 mM Tris–HCl buffer pH 7.0. DNA and prodigiosin were dissolved in the buffer at a concentration of 250 μg/mL respectively. Fluorescence spectra were recorded using an excitation wavelength of 480 nm and the emission range set between 480 and 600 nm using a slit width of 5/5 nm (Varioskan Flash Multimode Plate Reader, Thermo Scientific).

## Results

### Identification and quantification of prodigiosin

**Fig. 1:**
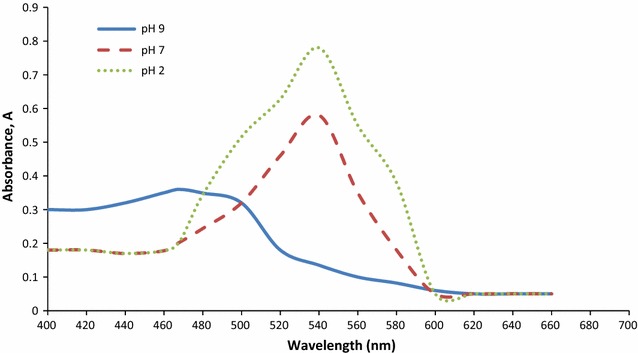
UV–Vis spectrum of prodigiosin at pH values of 2, 7 and 9 between 800 and 200 nm. The spectral pattern of the pigment was elucidated at pH values of 2, 7 and 9 between 800 and 200 nm, using methanol as blank

UV–vis analysis spectra for the red pigment in methanol showed a maximum peak at 535 nm. The red pigment changed to pink at pH 2, orange at pH 9 while retained its red colour at pH 7. Maximum absorbance for the pigment was recorded at λ_max_ 540 nm at pH 2.0, 533 nm at pH 7 and 468 nm at pH 9 (Fig. 1). The LC–MS spectrum showed a molecular ion peak of pigment at m/z 324 (prodigiosin has a molecular weight of 323). NMR analysis illustrated ^1^H-NMR. ESI, m/z 324.4 (M + H)+.

Solid state fermentation medium that contained peptone and glycerol was found to be a suitable medium for prodigiosin production. 947.57 prodigiosin unit/cell was produced under optimized conditions by *S. nematodiphila*. (Data not shown) Prodigiosin was also quantified using auto fluorescence method, and the concentration was found to be 50 mg L^−1^.

### Antimicrobial activity of prodigiosin

**Fig. 2:**
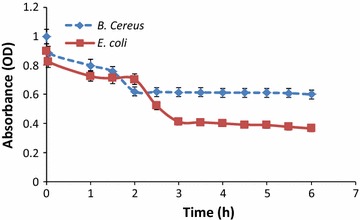
Kill curve showing bacteriostatic effect of prodigiosin on *Escherichia coli* and *Bacillus cereus*

It was found that prodigiosin had both bactericidal effects on both gram-positive and gram-negative pathogenic bacteria, *S. aureus* showed a zone inhibition diameter of 16.5 ± 0.42 (mm); *B. cereus* showed a zone inhibitor diameter of 10.5 ± 0.24 (mm); *P. aeruginosa* showed a zone inhibitor diameter of 12.5 ± 0.47 (mm); *E. coli* showed a zone inhibitor diameter of 11.5 ± 0.36 (mm).The turbidity assay where the bacteria after treatment with prodigiosin were measured at 600 nm, indicated that prodigiosin at concentrations up to 12 μg ml^−1^appeared to be bacteriostatic (Fig. 2). However at higher concentrations the pigment had a bactericidal effect.

### Determination of MIC and MBC

MIC for bacteria was in the range of 5–30 μg ml^−1^, MBC appeared to have different concentrations for different bacteria studied. It was calculated for *P. aeruginosa* (DT CT1): 16 µg/ml; *E. coli* (MTCC 729): 20 µg/ml; *S. aureus* (MTCC 96): 9 µg/ml and *B. cereus* (MTCC 1272): 12 µg/ml.

### Mode of bactericidal action of prodigiosin

#### Generation of ROS in prodigiosin treated bacterial cells

**Fig. 3:**
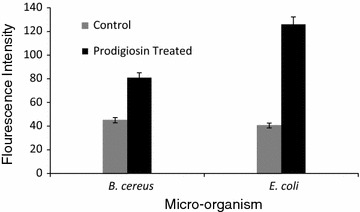
Measurement of ROS generated in prodigiosin treated bacterial cells. ROS level was measured with DCFH-DA fluorescence method as described before and analyzed by florescence Micro plate reader. Generation of ROS were more in gram negative than that of gram positive bacteria

The DCF fluorescence intensity was measured and is expressed with respect to control. A significant increase in ROS level was observed for both prodigiosin treated *B. cereus* and *E. coli* cells. Compared to the untreated control cells and prodigiosin treated *B. cereus*, enhanced generation of intracellular ROS was observed in *E. coli* treated with prodigiosin (Fig. 3).

#### Scanning electron microscopy (SEM)

**Fig. 4:**
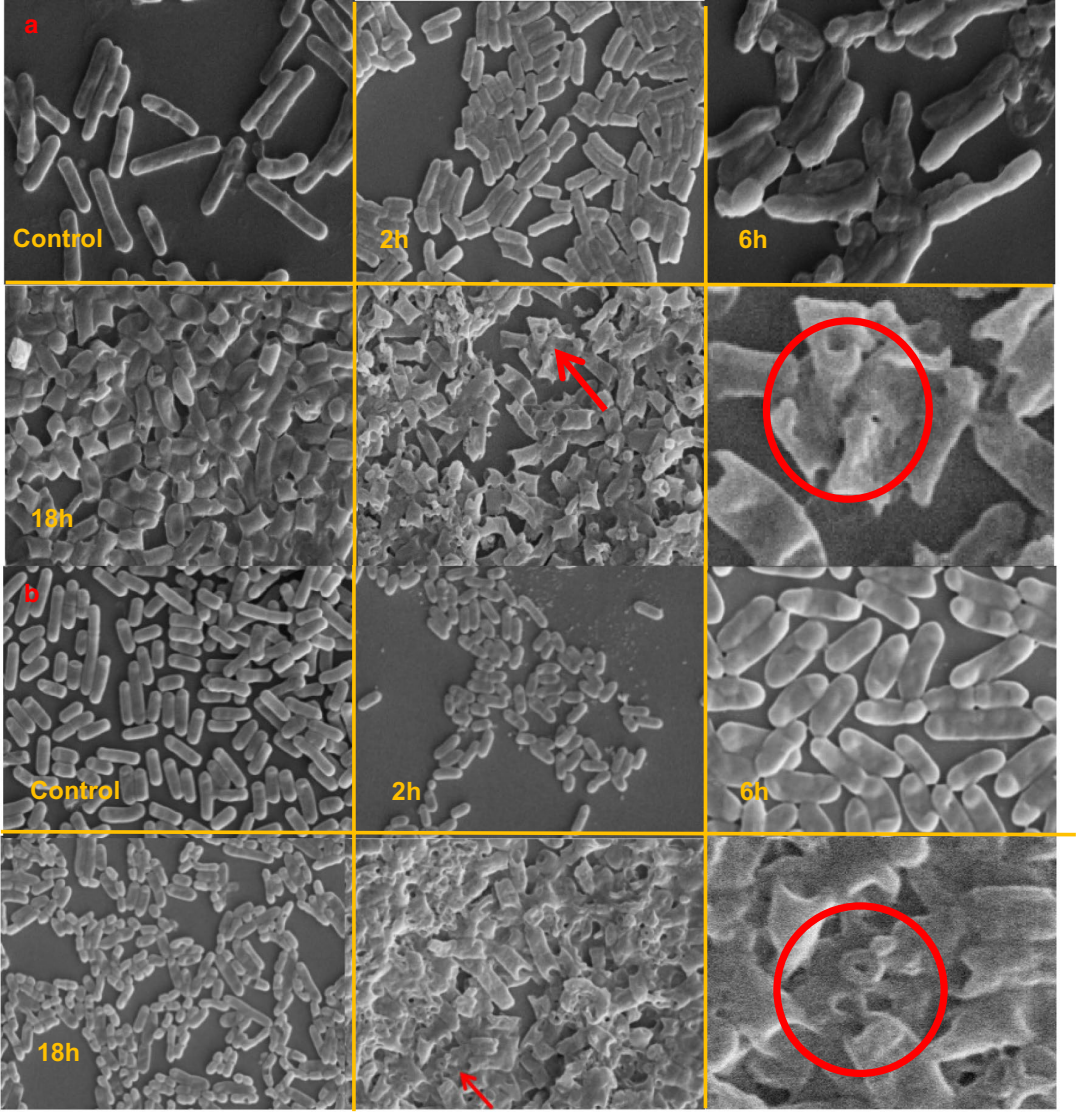
**a** and **b** Scanning electron micrograph of *Escherichia coli* and *Bacillus cereus.* Prodigiosin (50 µg/ml) was added to the test organism and were then incubated for* 2*,* 6*,* 18* and* 24* h at 37 °C. The cell pellet was washed twice with potassium phosphate buffer (0.01 M; pH 7.0) followed by overnight fixing of the pellet with 2 % glutaraldehyde. Then, the pellet was dehydrated in ethanol on a gradient mode (10–100 %). Upon completion of the wash with 100 % ethanol, the pellet was spread on to the previously sterilized slide as a thin uniform layer and the slides were dried in a desiccator for SEM observation

The prodigiosin treated bacteria showed an increase in size followed by the death of cells by cell membrane rupture and there was a formation of hallow on the outer membrane of bacterial cells (Fig. 4a, b). SEM also displayed a loss of membrane integrity at later stages. This was observed in both gram positive and gram negative bacteria studied. This result can be related to the breakdown of typical membrane bio dynamics and lysis that might be the primary mode of action.

#### Prodigiosin induce phosphatidylserine (PS) exposure at the outer membrane

**Fig. 5:**
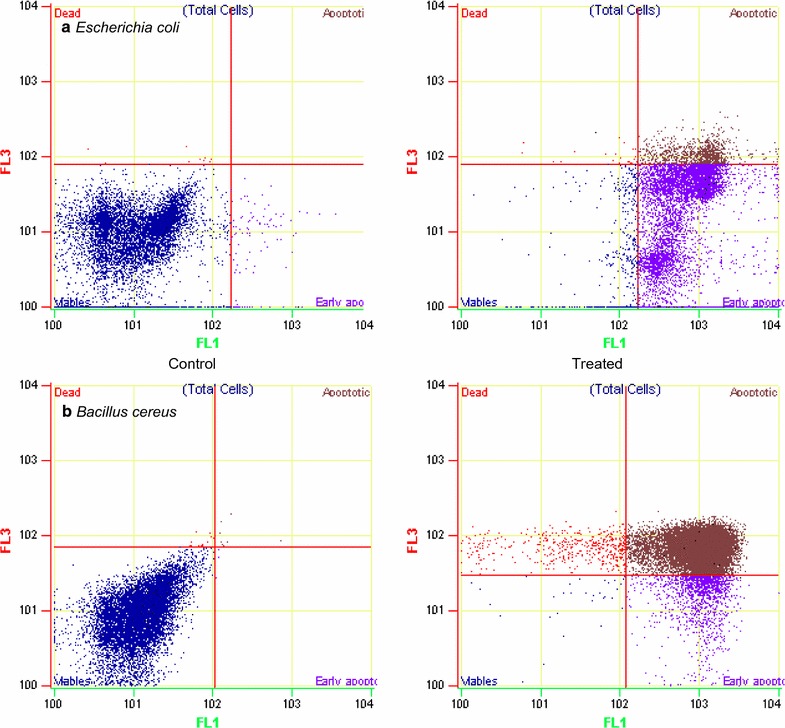
Analysis of Phosphatidylserine Exposure in *Escherichia coli* (**a**) and *Bacillus cereus* (**b**). The cells after prodigiosin treatment were resuspended in binding buffer and annexinV–FITC and PI were added according to Kit’s protocol and measured using Beckman coulter flow cytometer

Prodigiosin treatment proved to be the most proficient in inducing detectable PS exposure. In gram-positive bacteria, *B. cereus* there was 85.59 % apoptosis-like death. Also, significant numbers (>70 %) of Annexin V-positive *E. coli* were detected in prodigiosin treated cells. This result can be related to the breakdown of typical membrane bio dynamics and lysis that might be the primary mode of action which was also observed in SEM. Annexin V-positive cells in our study displayed a loss of membrane integrity at later stages (monitored by staining with the fluorescent intercalating dye, propidium iodide [PI], which requires structural permeabilization for detection), demonstrating that PS exposure was primarily detected at the outer membrane; cells that are both annexin V- and PI-positive were considered to be dead (Fig. 5).

#### Analysis of depletion of the lipopolysaccharide (LPS) layer

**Fig. 6:**
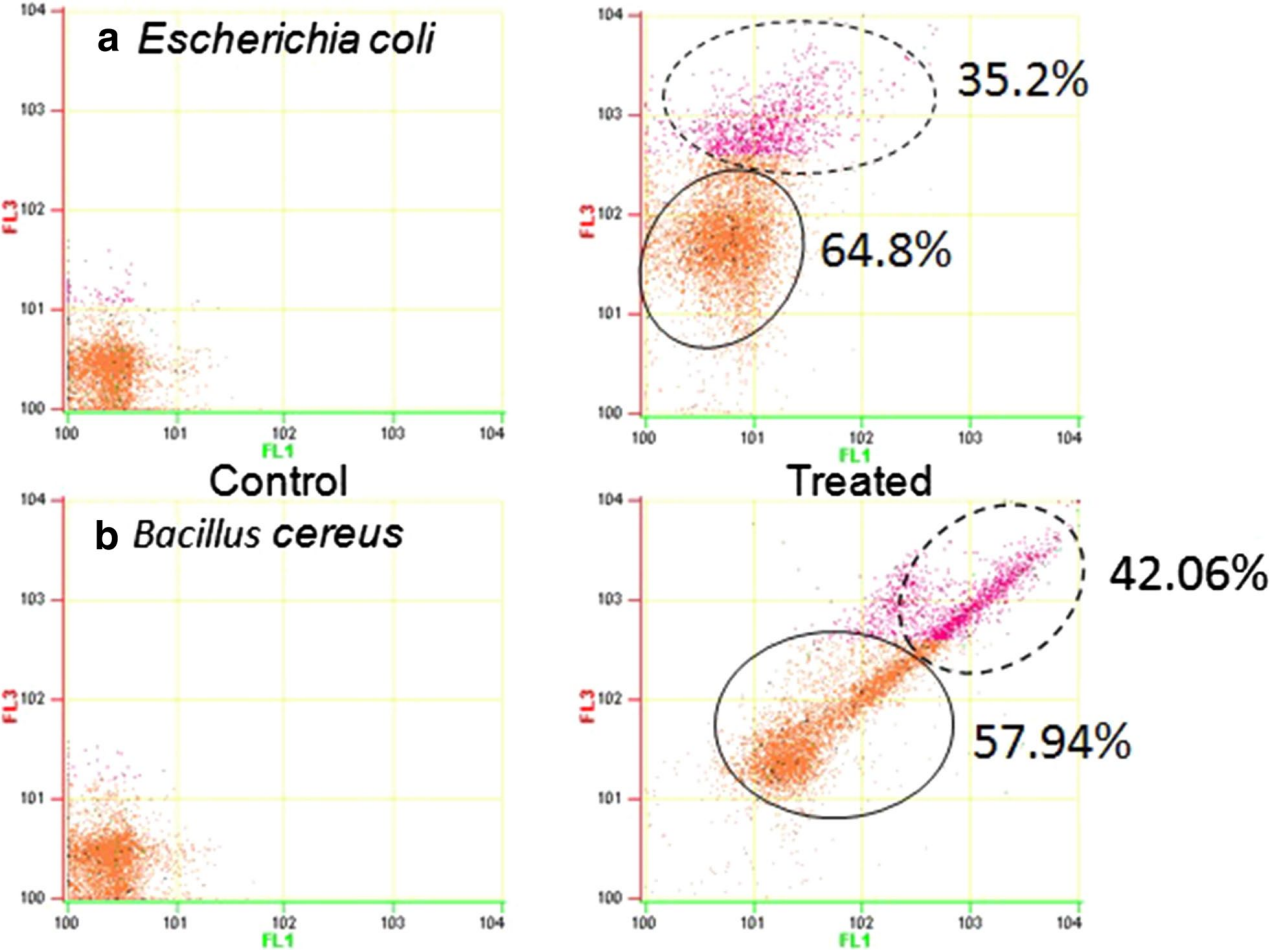
Analysis of depletion of the lipopolysaccharide (LPS) layer in *Escherichia coli* (**a**) and *Bacillus cereus* (**b**). The cells after prodigiosin treatment were resuspended in binding buffer and TO and PI were added and measured using Beckman coulter flow cytometer

Control cells were impermeable to propidium iodide (PI) due to the fact that these cells have intact membranes, Both untreated and prodigiosin treated cells were permeable to thiazoleorange (TO) on varying degrees, PI is permeable in only those cells whose membrane is damaged, Depletion of the lipopolysaccharide layer greatly facilitates TO uptake (Torres et al. 2012). The TO and PI dye combination provides significant resolution between live and dead cell populations (Fig. 6) prodigiosins being lipophilic in nature are, very unstable in water solutions, and might diffuse freely through membranes.

#### Motility test for prodigiosin treated pathogens

**Fig. 7:**
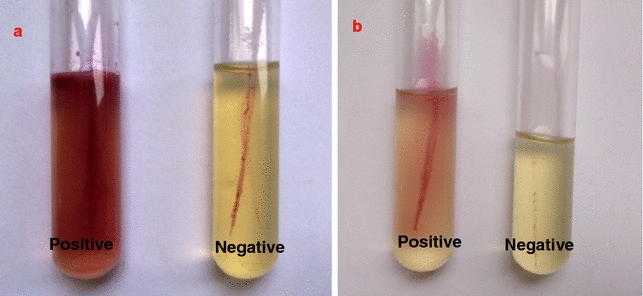
Motility Test medium tubes containing TTC inoculated with *Escherichia coli* (**a**) and *Bacillus cereus* (**b**). Positive before treatment negative after treatment with prodigiosin

In agar stab method, the control cells migrated readily throughout the tube, causing red coloured cloudiness indicated by the reduction of TTC to formazon, the prodigiosin treated bacteria showed red coloured formation only in the area where they were inoculated. Prodigiosin treated flagellated pathogens doesn’t showed a well dispersed growth from the line of inoculation which is evident for lack of motility, when compared with the non-treated control cells which spreads out from the line of inoculation and had grown throughout the medium (Fig. 7).

#### Expression of a protein with caspase-like substrate specificity

**Fig. 8:**
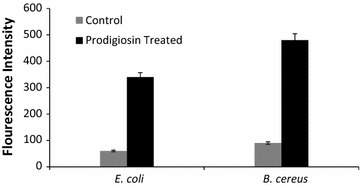
Analysis of expression of a protein with caspase-like substrate specificity in prodigiosin treated bacterial cells. Detection of protease activity in bacterial cells using the EnzChek Caspase-3 Assay Kit #2 with Z-DEVD–R110 substrate. Cells were either treated with prodigiosin for 4 h at 37 °C to induce apoptosis (induced) or left untreated (control). Both induced and control cells were then harvested, lysed and assayed as described in the kit protocol. Reactions were carried out at room temperature and fluorescence was measured in a fluorescence microplate reader using excitation at 485 ± 10 nm and emission detection at 530 ± 12.5 nm after the indicated amount of time

In our studies, we observed expression of caspase- like substrate specificity in prodigiosin treated bacterial cells (Fig. 8). The bisamide derivative of rhodamine 110 (R110) which contained DEVD peptides covalently linked to each of R110’s amino groups, suppressed the dye’s visible absorption and its fluorescence. Upon enzymatic cleavage, the nonfluorescent bisamide substrate is converted in a two-step process first to the fluorescent monoamide and then to the even more fluorescent R110. Both of these hydrolysis products exhibit spectral characteristics similar to those of fluorescein, with peak excitation and emission wavelengths of 496 nm and 520 nm, There was 5.6-fold higher concentration of caspase like proteases in *E. coli* and 5.3-fold higher in *B. cereus.*

#### DNA fragmentation assay

**Fig. 9:**
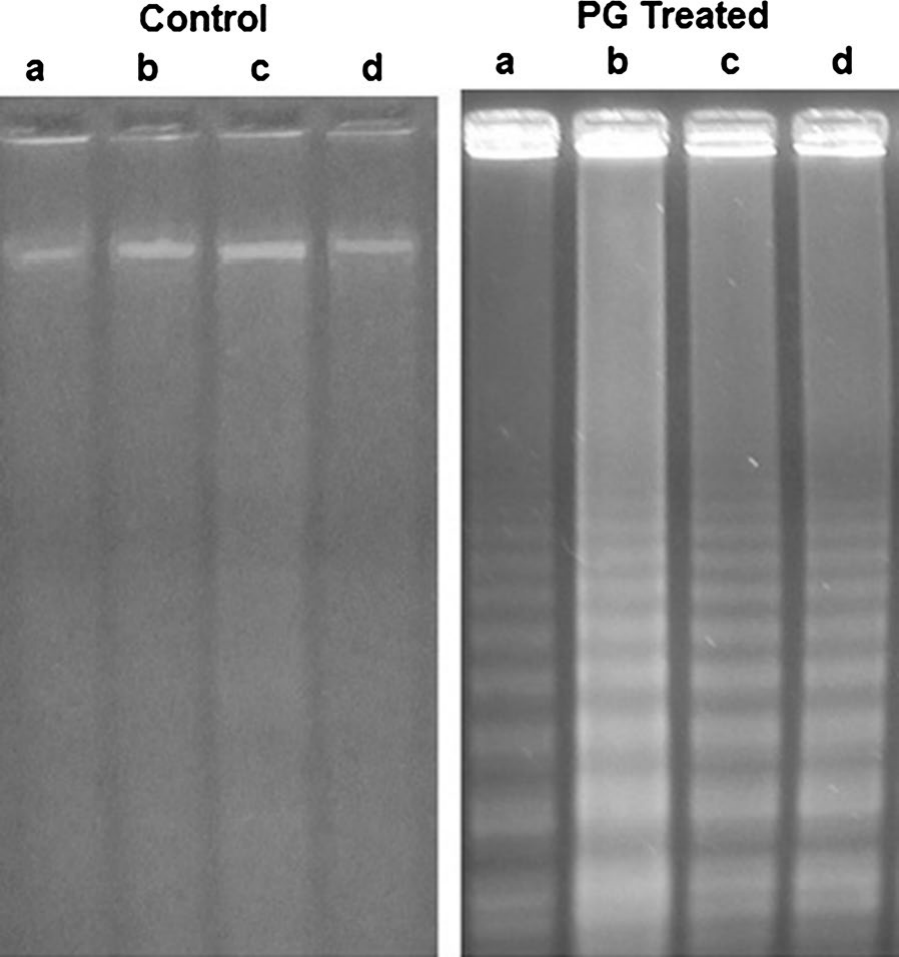
DNA fragmentation assay showing ladder like formation in DNA extracted from *a* *Bacillus cereus*(MTCC 1272), *b* *Staphylococcus aureus* (MTCC 96), *c* *Pseudomonas aeruginosa*(DT CT1) and *d* *Escherichia coli* (MTCC 729)

The DNA damage was observed in bacterial cells incubated overnight in the presence of 50 μg ml^−1^ of prodigiosin (Fig. 9). Agarose gel electrophoresis of DNA showed the characteristic ladder pattern. Prodigiosin treated bacterial cells exhibited extremely high DNA damaging effects.

## Discussion

### Identification and quantification of prodigiosin

Prodigiosina red pigment synthesized by *Serratia sp.* is a secondary metabolite that belongs to the family of tripyrrole from *S. nematodiphila* induces programmed cell death like activity against few food borne bacterial pathogens. Our results have established the biochemical and physiological changes that are apparent during bacterial cell death. The tomato plant isolate producing red pigment was identified by morphological, biochemical and genotypic analysis as *S. nematodiphila*. This is the first report of *S. nematodiphila* producing antimicrobial prodigiosin.

In *S. nematodiphila*, the pigment precipitated out from within the cells and colonies changed from red to dark maroon by 36 h of incubation, often with a metallic green sheen under reflected light. At this stage, most cells in the colony were no longer viable. Prodigiosin production by *S. nematodiphila* in peptone glycerol agar plates was quantified. The produced prodigiosin pigment can be qualitatively and quantitatively analysed by using the autofluorescence nature of the prodigiosin and can be utilized as a new tool for the quantification of prodigiosin.

### Antimicrobial activity of prodigiosin

Prodigiosin showed significant antimicrobial activity against *P. aeruginosa*, *E. coli, S. aureus and B. cereus* (Fig. 2). The antibacterial activity of prodigiosin (PG) is the result of their ability to pass through the cellular membrane and damaging it and their capacity for inhibiting target enzymes involved in DNA replication, such as topoisomerase IV and DNA gyrase, which inhibit the cell growth (Berlanga et al. 2000). Prodigiosin due to its hydrophobic properties is proposed to influence the function of biological membranes. Prodigiosin can enter into the cytoplasm of bacterial cells and, at higher concentrations, affects the membrane integrity by depletion of lipopolysaccharide layer of *E. coli* and *B. cereus*. Prodigiosin could be a replacement of natural pigments to synthetic colorants for possible application in the food industry (Darshan and Manonmani 2015).

### Mode of bactericidal action of prodigiosin

#### Generation of ROS in prodigiosin treated bacterial cells

The redox state of the cell is primarily a consequence of the production and elimination of ROS. ROS generation was found to be higher for gram-negative bacterial cells as compared to that for gram-positive bacterial cells (this study). Positively charged prodigiosin might have enhanced the production of ROS upon its interaction with negatively charged bacterial membrane, resulting in higher toxicity for *E. coli* (Arakha et al. 2015). The lipid peroxidation in bacterial membrane might have led to ROS generation. Prodigiosin may effectively enter bacterial cells and promote induction of intracellular level of reactive oxygen species. The production of ROS could have played a major role in the bacterial cell death. ROS are necessary for the various physiological activities of cells, but an imbalance for reactive oxygen species results in increased oxidative stress which may damage a variety of macromolecules. ROS may lead to loss of function, an increased rate of mutagenesis, and ultimately cell death (Cabiscol et al. 2000).

#### Scanning electron microscopy (SEM)

Prodigiosins has an affinity towards the lipids bilayer of the plasmatic membrane because C-pyrrole ring of prodigiosin is lipophilic due to the presence of pentyl chain. Prodigiosin had been reported to generate lipid peroxidation in the presence of Cu (II) and damage cytoplasmic membrane in its cytotoxicity(Subramanian et al. 2007). We propose that lipid peroxidation corresponds to the ultimate disintegration of membrane integrity. The prodigiosin, linked to the bacterial plasma membrane, could captivate visible light and generate reactive oxygen. The pigment and the cellular membrane were attacked by reactive oxygen. Subsequent lipid peroxidation damaged the integrity of the cellular membrane (Wang et al. 2013)that could be clearly observed in the SEM (Fig. 4a, b). The same results were reflected in the analysis of depletion of lipopolysaccharide layer.

#### Analysis of depletion of the lipopolysaccharide (LPS) layer

LPS molecules are electrostatically accompanied by divalent cations (e.g., Mg^2+^ and Ca^2+^). This structure can be weakened by removing divalent ions or replacing them with other cationic agents. Prodigiosin being cationic in neutral pH further results in an increase of outer membrane permeability and sensitizes the bacteria to hydrophobic prodigiosin molecule. As LPS acts as a key virulence factor, some studies have pointed out that modification of lipid A of LPS is a key component of adaptive antimicrobial resistance (Lam et al. 2011). Effect of prodigiosin on LPS makes it a substantial antimicrobial agent.

#### Motility Test for prodigiosin treated pathogens

These results suggest that the flagella are underdeveloped causing lack of motility. Prodigiosin might Effect Intracellular pH of bacteria that in turn result in variation of rotational speed of bacterial flagellar motors (Minamino et al. 2003).

This indicated the effect of prodigiosin in bringing down the motility capacity which is very essential factor for pathogenicity. As the swarming capacity reduces, the availability of nutrients also reduces and hence there has been a bactericidal effect. The flagella formation was inversely related to the pigment production (Kobayashi and Ichikawa 1990).

#### Expression of a protein with caspase-like substrate specificity

Caspases are regarded as the critical regulators of programmed cell death in metazoans (Thornberry and Lazebnik 1998). The prodigiosin treatment resulted in expression of caspase like proteins with protease activity, which played an important role in bringing out programmed cell death in bacterial cells. While distant, sequence-based relatives of caspase-like proteins (e.g., meta- and paracaspases) have been recognized in silico in various unicellular organisms, however, bacterial orthologs have yet to be ascertained based on DNA sequence (Vercammen et al. 2007).

#### DNA fragmentation assay

In order to determine whether bacterial cell death was analogous to programmed cell death, we analysed induction of DNA fragmentation by prodigiosin. DNA damage mechanisms involving oxidative damage may contribute to the programmed cell death like activity against bacterial pathogens.

For prodigiosin, an addition of DNA caused a 14 nm bathochromic shift from 533 to 547 nm. Fluorescence enhancements were also observed for molecules upon DNA binding, with changes in the emission which were much more dramatic for DNA binding by prodigiosin. Prodigiosin is colored deeply red due to the planar pyrrolyl pyrromethene chromophore and is cationic at neutral pH. Determination of these facets of prodigiosin chemistry would facilitate an understanding of their mode of action in a biological system (Melvin et al. 1999). The fluorescent properties of Prodigiosin permitted to gain insight into their mode of DNA binding. There was a change in spectral band position in the emission spectrum of prodigiosin molecule to a longer wavelength (lower frequency). We then utilized fluorescence spectroscopy to study DNA binding by prodigiosin, Addition of excess DNA led to an increase in the intensity of emission for prodigiosin. Le Pecq and Paoletti (1967) have shown that the energy of UV absorbance by DNA (donor) base pairs may be efficiently transferred to an intercalated fluorophore (acceptor).The C-pyrrole ring of prodigiosin is lipophilic due to the pentyl chain, and as discussed by Hecht and co-workers (1988), lipophilic groups bind DNA through interaction with the least polar component of the duplex, i.e., with the interior.

**Fig. 10:**
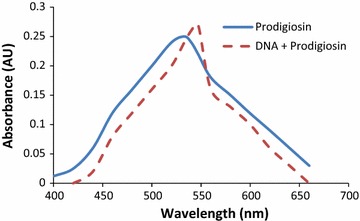
The 14 nm shift in the fluorescence pattern is an indicative of the intercalation of DNA with prodigiosin

Our interest in the 4-methoxypyrrolic natural products has been focused on gaining an understanding of their DNA-targeting properties. The shift in the fluorescence pattern is an indicative of the intercalation of DNA with prodigiosin (Fig. 10). The planar prodigiosin nucleus may bind DNA by intercalation while the methoxy group and ring nitrogen provide hydrogen-bonding sites to facilitate DNA binding. The cationic nature at neutral pH also provides electrostatic interaction with the negative phosphate groups of the DNA helix.

In conclusion, our studies have revealed that bacterial death involves DNA fragmentation, Phosphatidylserine exposure and expression of a protein with caspase-like substrate specificity. Prodigiosin at sub-inhibitory concentrations inhibits motility of pathogens which may be due to either loss of flagella or loss of invasive capacity. In conclusion, the present results show for the first time that sub-inhibitory concentrations of prodigiosin inhibit motility of bacterial cells. As flagella-mediated motility is required for virulence, and the colonization, and at higher concentration prodigiosin effect could be facilitated through a Programmed cell death like phenomenon.
